# Dynamic maps of plastic-mulched farmlands in Northeast China from 1985 to 2025

**DOI:** 10.1038/s41597-026-07400-2

**Published:** 2026-05-06

**Authors:** Bowen Niu, Xinmin Zhang, Yifang Zhang, Xiaolu Yan, Dehai Zhu, Quanlong Feng

**Affiliations:** 1 College of Land Science and Technology, China Agricultural University, Beijing, 100193 China; 2 Laboratory of Geo-Information Science and Remote Sensing, Wageningen University & Research, 6708 PB Wageningen, The Netherlands

## Abstract

We provide PMF-NEChina-40D, the first plastic-mulched farmlands (PMFs) dynamic monitoring dataset in Northeast (NE) China for the past four decades from 1985 to 2025. Multi-sensor Landsat series and Sentinel-2 imagery were processed on the Google Earth Engine platform, and a dual-temporal PMFs mapping framework integrating *film-on* and *film-off* stages was proposed. Specifically, the Random Forest classifier, combined with grid-based partitioned modeling, was used to produce PMFs maps with spatial resolutions of 30 m (1985–2015) and 10 m (2020–2025) for nine time steps and every five years, including 1985, 1990, 1995, 2000, 2005, 2010, 2015, 2020 and 2025. Training and validation samples were generated through visual interpretation supported by multi-source satellite imageries and field surveys. Classification accuracy was evaluated using multiple metrics, with average user’s accuracy, producer’s accuracy, F1 score and overall accuracy exceeding 90%. The resulting dataset yields the first long time-series PMFs distribution maps of Northeast China with high spatial resolution across the past four decades. The dataset offers a reusable resource for studies of agricultural land management, environmental assessment and sustainable development in Northeast China black soil region, and can support applications in other regions with similar agricultural practices.

## Background & Summary

Plastic mulching is a key agricultural technique that significantly improves the crop growth environment by laying plastic films, providing multiple benefits such as heat preservation, soil moisture retention, weed suppression, and yield enhancement^1,2^. China is the largest consumer of plastic mulch films worldwide, accounting for approximately 75% of global usage^3^. Against this backdrop, Northeast China black soil region—one of the four major black soil regions in the world and regarded as the “panda of arable land” due to its deep soil layer, rich organic matter, and strong nutrient-retention capacity—stands out as China’s most important high-quality farmland resource^4,5^. As a major national grain-producing area, Northeast China has witnessed the rapid adoption of plastic mulching in recent years, driven by the growing economic returns of staple crops such as maize and soybean. According to statistics, the use of plastic mulch films in the Northeast China black soil region increased from about 100,000 tons in 2010 to about 200,000 tons in 2020, with an average annual growth rate of 7.2% *(National Data:* https://data.stats.gov.cn/index.htm*)*.

Currently, the plastic mulch films widely used in agricultural production are mainly made of polyethylene (PE), a material that is extremely resistant to degradation under natural conditions^6,7^. Due to the long-term and large-scale application of mulching films and inadequate recycling, residual films accumulate in soils, leading to increasingly severe pollution problems^8,9^. Studies have shown that residual films can damage soil structure, hinder water infiltration and gas exchange, and even induce secondary salinization, posing serious threats to the ecological environment and sustainable agricultural development of the Northeast China black soil region^10,11^. Therefore, long time-series monitoring of PMFs distribution to reveal their spatiotemporal dynamics are of great importance for protecting black soils and promoting sustainable agricultural development. In this study, PMFs refer to croplands where plastic films are directly applied on the soil surface during crop growth to regulate soil temperature and moisture. PMFs are distinct from plastic-covered greenhouses (PCGs), which are facility-based agricultural systems with fixed or semi-fixed structures. This study focuses on white plastic films, as they exhibit stable and high reflectance in optical imagery, making them suitable for large-scale and long-term PMFs mapping. In contrast, black or dark films are not considered due to their visual similarity to farmlands and shadows, which makes them difficult to be reliably identified.

Traditional surveys of PMFs have mainly relied on field inspections and quadrat statistics, which can ensure a certain level of accuracy but remain limited in terms of efficiency, spatial coverage, and timeliness^12,13^. With the advancement of remote sensing technology, researchers have explored various PMFs mapping methods. For instance, spectral-index based approaches have been adopted to extract PMFs by constructing specific plastic spectral indices^14^ (e.g., PMLI). These methods are simple and do not require large training samples, but are influenced by regional spectral variability, making it difficult for large-scale and long-term PMFs mapping. In contrast, classification methods based on local computing servers require substantial computational and storage resources^15,16^. Traditional machine learning algorithms such as Random Forest (RF) and Support Vector Machine (SVM) can integrate spectral, textural, and temporal features to improve PMFs mapping performance^17^. For instance, Hasituya *et al*. employed Landsat-8, GF-1, and Radarsat-2 imagery to extract PMFs in Zhuozhou, Hebei Province, and Guyuan, Ningxia^18–21^. Additionally, deep learning methods (e.g., UNet) can automatically capture high-dimensional and multi-scale spatial–spectral features to improve PMFs classification accuracy^22–24^. For example, Wei *et al*. applied a semantic segmentation model based on Beijing-2 satellite imagery to identify PMFs in parts of Gansu and Xinjiang^25^. However, both classic machine learning and deep learning approaches generally rely on substantial local computational power and storage capacity, which limits their efficiency and scalability for large-scale and long-term PMFs mapping. As a result, most existing studies are confined to small regions, short time spans, or specific crop/mulching types, and thus fail to capture the long-term dynamics of PMFs at regional to decadal scales.

In comparison, Google Earth Engine (GEE) offers significant advantages for processing and managing multi-temporal, large-scale datasets^26,27^. Without the need for high-performance local hardware, GEE enables rapid remote sensing image classification, providing an efficient pathway for large-area land-use/land-cover (LULC) mapping^28,29^. For example, Feng *et al*. combined GEE with a Random Forest classifier to produce a national-scale plastic-covered greenhouses (PCGs) distribution map for 2019^30^, and Zhang *et al*. used GEE to generate multiple LULC datasets, including wetlands, impervious surfaces and other datasets^31–34^. Nevertheless, large-scale and long-term PMFs mapping remains challenging. Firstly, PMFs’ spectral characteristics vary with crop growth and are easily confused with other plastic-covered surfaces such as PCGs. In addition, substantial regional differences in environmental conditions and cropping patterns restrict the transferability of spectral indices, limiting their effectiveness for long-term and large-area PMFs mapping. Reflecting these challenges, most PMFs classification studies only focused on small areas and limited periods^35–37^, only Xiong *et al*. developed a GEE-based framework using Landsat-5/7/8 and Sentinel-2 to generate multi-year PMFs maps in Xinjiang^38^. As a result, long-term spatio-temporal PMFs datasets for the Northeast China black soil region remain scarce, hindering a comprehensive understanding of the evolution of mulching agriculture in this area.

To bridge this gap, we developed a PMFs extraction framework on the Google Earth Engine (GEE) cloud platform by integrating both spectral and phenological features derived from multi-temporal remote-sensing imagery. The framework incorporates a dual-phase (i.e., *film-on* and *film-off*) identification strategy, which leverages temporal variations in surface reflectance to discriminate PMFs from other land objects, particularly those with high-reflectance such as plastic-covered greenhouses (PCGs) and bare land, thereby reducing misclassification. Using this framework, we produced the first long-term series of PMFs maps for Northeast China covering the past four decades (1985–2025), termed PMF-NEChina-40D^39^. This dataset, the first of its kind, integrates observations from multiple sensors across a vast agricultural region, providing a robust foundation for investigating the spatio-temporal evolution of mulching agriculture in the Northeast China black soil region.

**Fig. 1:**
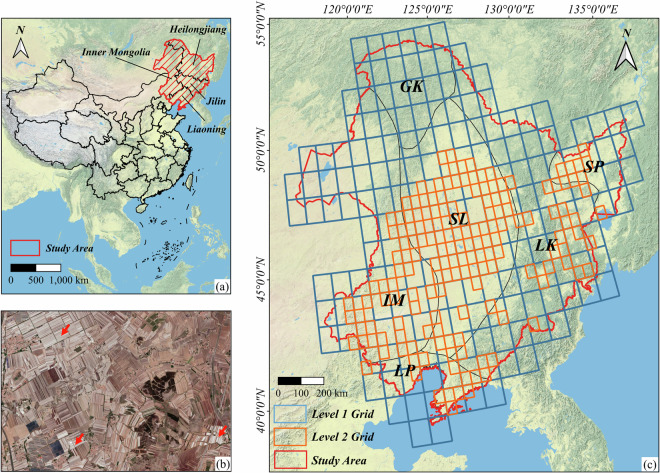
Study area. (**a**) relative location of the study area within China; (**b**) appearance of PMFs in satellite imagery where red arrows indicate PMFs; (**c**) study-area map. Following the Regionalization of Agro-climate of China^54^, Northeast China is partitioned into six agro-climatic zones, including North Greater Khingan (GK), Sanjiang Plain (SP), Lesser Khingan–Changbai Mountains (LK), Songliao Plain (SL), Liaodong Peninsula (LP), and East Inner Mongolia (IM). The red outline denotes the study area boundary. The blue and orange grids represent the Level-1 and Level-2 grids used for classification and data organization, respectively; the Level-2 grids correspond to the year 2025. Projection: Albers Equal Area Conic projection.

Above all, we provide the first long-term dataset of PMFs in Northeast China black soil region from 1985 to 2025 with high spatial resolution of 30-m and 10-m. The study area, encompassing Heilongjiang, Jilin, Liaoning Provinces and four prefectures in eastern Inner Mongolia, is shown in Fig. 1, and the proposed dataset could be of particular interest to the following research areas.

### Plastic pollution monitoring

The proposed dataset allows assessment of the spatial extent and dynamics of agricultural plastic usage, supporting evaluations of residual films accumulation and its environmental impacts in Northeast China.

### Ecological environment evaluation

The spatially explicit PMFs maps provide a basis for analyzing interactions between mulching agriculture, water cycles, and ecosystem health.

#### Agricultural policy-making

The proposed dataset offers quantitative evidence for formulating and assessing regional policies on mulching film management, agricultural modernization, and green transformation.

#### Black soil protection strategies

The proposed dataset supports the design and monitoring of targeted measures for conserving the fertility and sustainability of the Northeast China black soil region.

#### Agricultural non-point source pollution assessment

The dataset provides spatial inputs for modeling nutrient and pollutant runoff associated with mulched farmlands.

## Methods

### Study area

This study focuses on the Northeast China black soil region, which includes Heilongjiang, Jilin, and Liaoning provinces, as well as four prefectures in eastern Inner Mongolia (Hulunbuir, Hinggan League, Tongliao, and Chifeng)^40^ (Fig. 1). The region encompasses the Songnen Plain, Sanjiang Plain, and Liaohe Plain. It is characterized by flat terrain and fertile soils, making it suitable for large-scale, contiguous farmland development. Located in the temperate monsoon climate zone, the region receives an average annual precipitation of about 400–650 mm, most of which occurs during the growing season, and supports a stable single-cropping system dominated by spring-sown crops. The region is renowned for its highly productive soils dominated by Chernozems (black soils) and Phaeozems (black calcareous soils). The black soil layer is typically 30–70 cm thick, with organic matter contents ranging from 3% to 10%^41^. As one of China’s most important grain-producing bases, the region contributes about one-quarter of the nation’s total grain output. In addition, to further enhance productivity, plastic mulching practices are widely applied in the cultivation of major crops such as maize and soybean^42^.

### Overall workflow

**Fig. 2:**
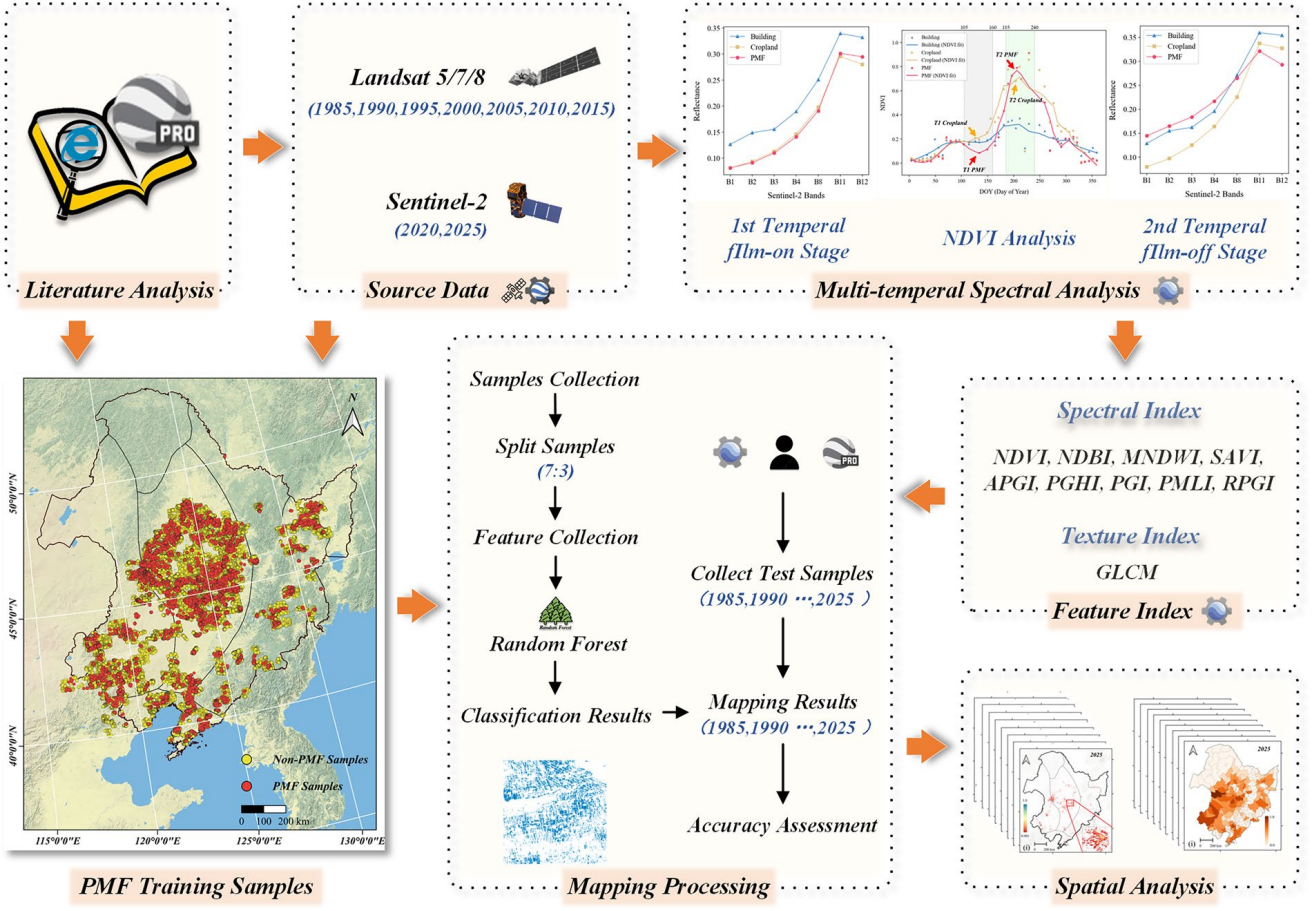
Flowchart.

This study was conducted on the GEE cloud platform using multi-temporal imagery from Landsat-5/7/8 and Sentinel-2 (Fig. 2). Based on field survey and a systematic literature review, together with an in-depth analysis of the spectral and phenological features of PMFs, we generated multi-year training samples from both Google Earth Pro historical imagery and GEE. For each year, we constructed a multi-temporal and multi-dimensional feature set comprising original spectral bands, spectral indices and texture features, and applied a Random Forest classifier to achieve high-accuracy PMFs mapping for Northeast China over the past 40 years. For accuracy assessment, we combined “online” (GEE) and “offline” (Google Earth Pro) procedures to assemble independent test samples for each year and evaluated the PMFs mapping performance using multiple accuracy metrics. The resulting dataset, PMF-NEChina-40D, provides reliable support for analyzing the spatio-temporal dynamics of PMFs in Northeast China and for related downstream research.

### Data source and data pre-processing

#### Data source

For satellite remote sensing data, we selected multi-sensor imagery from nine representative years at five-year intervals over the past four decades (1985–2025) to systematically reveal the spatio-temporal evolution of PMFs in Northeast China. Considering historical data availability and image quality, Landsat-5 TM was used for 1985, 1990, and 1995; Landsat-7 ETM + for 2000, 2005, and 2010; Landsat-8 OLI for 2015; and Sentinel-2 MSI for 2020 and 2025. During the historical period (i.e., 1985–2015), PMFs mapping was primarily based on Landsat imagery (30 m), whereas Sentinel-2 data (10 m) were introduced for the more recent years (i.e., 2020 and 2025) because their higher spatial resolution allows better identification of small and fragmented PMFs patches. Meanwhile, to address the scan-line corrector (SLC) failure in Landsat-7 ETM + data, the SLC-off gaps were filled using multi-temporal neighboring observations^43,44^. Sentinel-2 data were employed for 2020 and 2025 due to their higher spatial resolution, which enables better identification of fine-scale PMFs patterns. Besides, the higher temporal resolution of Sentinel-2 could improve data availability during the short *film-on* period, which is critical for large-scale PMFs mapping. For cropland data, we employed the dataset GLC_FCS30D^31^, a global 30-m long-term LULC maps produced at five-year intervals for the period 1985–2022, which provides high temporal consistency and spatial continuity.

#### Data pre-processing

All satellite images were pre-processed on the GEE platform. Given the vast extent of Northeast China, directly classifying such a large area would impose computational constraints and efficiency bottlenecks. To improve data processing efficiency, the study area was first divided into 1° × 1° grids for data organization and management. Based on this framework, 0.5° × 0.5° grids were further constructed as the basic units for PMFs classification, which were used to perform partitioned modeling in PMFs mapping tasks. Since PMFs occurred only within croplands, we further utilized crop layer from GLC_FCS30D to filter the grids and retained only those containing cropland (Fig. 1). It should be noted that the cropland layer was used to constrain the spatial extent at the grid level rather than as a pixel-wise mask. Specifically, all pixels within grids containing cropland were classified, rather than restricting the analysis only to cropland pixels. As a result, some non-cropland objects may still be included within these grids. For cropland constraints, a combined dynamic and static strategy was adopted. For the period 1985–2019, cropland layers from GLC_FCS30D corresponding to each target year were used. For 2020–2025, the 2020 cropland layer was applied as a static mask because updated land-cover data for 2025 are not yet available. This approach effectively constrained the mapping extent while maintaining temporal consistency in PMFs mapping.

### Spectral & phenological characteristics analysis and feature selection

#### Spectral & phenological characteristics analysis

**Fig. 3:**
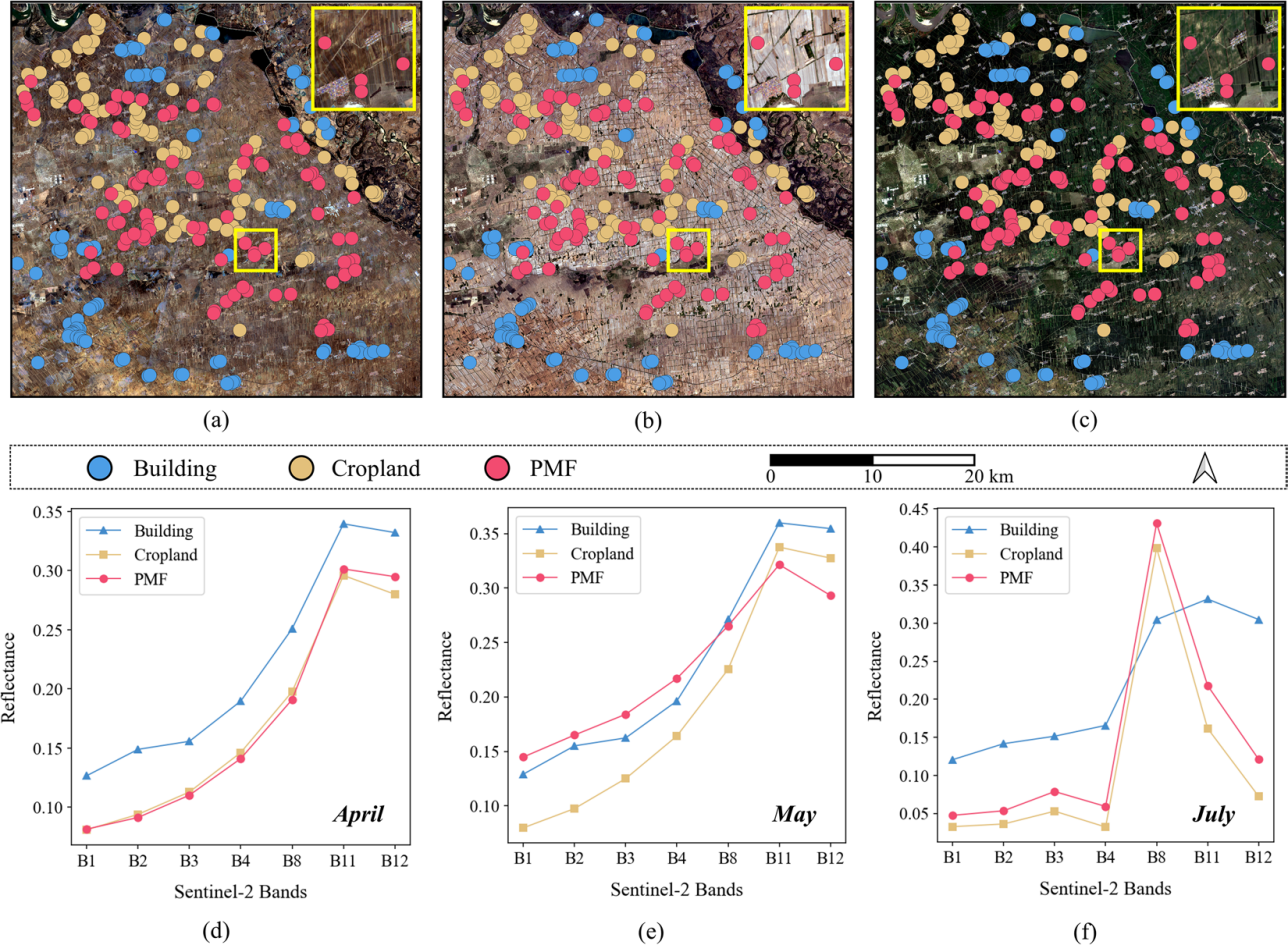
Spectral & phenological characteristics analysis. Note*. We used 2020 Sentinel-2 multispectral data to compare the spectra of PMFs and non-PMFs. Figure 3 a-d, b-e, and c-f show three matched pairs, including both satellite imagery with the corresponding reflectance spectra. The centroid of the study area is located at 45°14′15″N, 125°32′23″E.

**Fig. 4:**
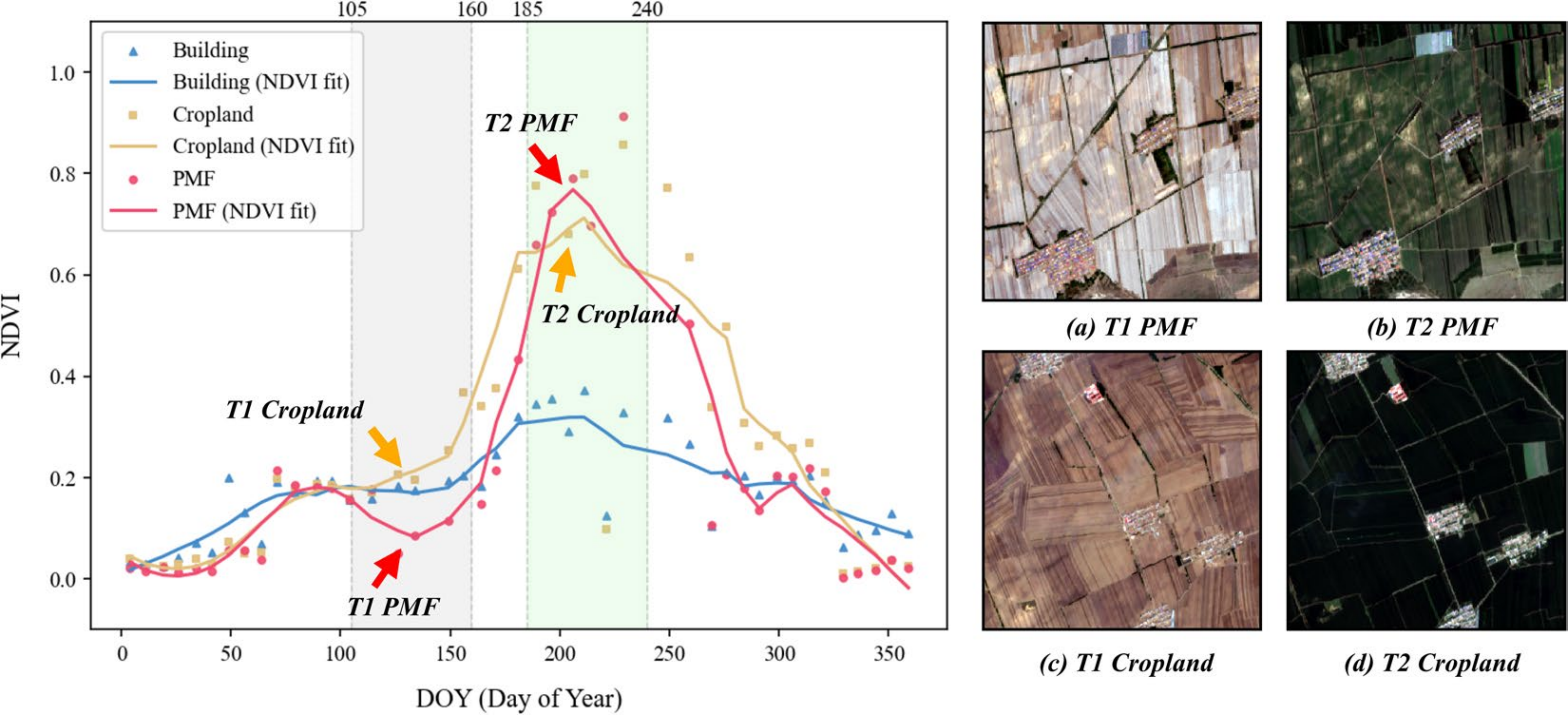
Time-series NDVI curves for PMFs and other land covers. T1 and T2 refer to *film-on* and *film-off* stages, respectively.

To investigate the spectral differences between PMFs and non-PMFs, we collected samples from 2020 Sentinel-2 imagery to derive spectral reflectance curves for key growth stages including April, May, and July (Fig. 3). Meanwhile, we also produce NDVI time-series profiles using the same samples to observe the phenological characteristics of PMFs (Fig. 4).

Figure 3 revealed that PMFs in Northeast China exhibit distinct spectral patterns along the crop growth period. During the sowing and mulching stage (April to May), when crops are still in their early growth phase and plastic mulch is clearly visible, PMFs show relatively high reflectance in the visible bands (B2, B3, B4) and generally exhibit low NDVI values (Fig. 4a,b).

At this *film-on* stage, PMFs appear bright white and are most easily identified. During the vigorous growth stage (around June), as the mulch films gradually degrade and farmlands are covered by crops, NDVI values for PMFs increase substantially, and crop reflectance dominates their spectral response. Although PMFs can still be distinguished from non-PMFs in the near-infrared and certain red-edge bands, the separability is significantly reduced. By the crop maturity stage (early July onward), the reflectance spectra and NDVI curves of PMFs and non-PMFs (e.g., cropland) just converge (Figs. 3f, 4), leading to a decline in between-class separability.

Because plastic mulch covers farmland for only a short period (approximately 1–2 months), PMFs extraction based on single-date imagery is easily affected by crop growth, cloud/rain conditions, and confusion with other highly reflective surfaces such as plastic-covered greenhouses. To address this issue, we analyzed the spectral evolution of PMFs and the typical cropping calendar of major spring crops (e.g., maize and soybean). By integrating agricultural statistics, scientific literature, and local agronomic reports, we developed a dual-temporal feature based PMFs mapping framework that combines both *film-on* and *film-off* stages. Specifically, the *film-on* stage (late April to early June) corresponds to crop sowing and emergence, when the plastic mulch covering farmland remains exposed, producing distinct spectral and textural features that make this period optimal for PMFs identification. The *film-off* stage (around August) corresponds to the crop maturity phase, when farmlands are fully covered by vegetation and the spectral response is dominated by crops other than plastic mulches beneath. Although not suitable for direct plastic mulch detection, the *film-off* stage is valuable for excluding high-reflectance surfaces such as greenhouses and bareland, thereby reducing false positives and improving PMFs mapping accuracy.

#### Feature selection

**Fig. 5:**
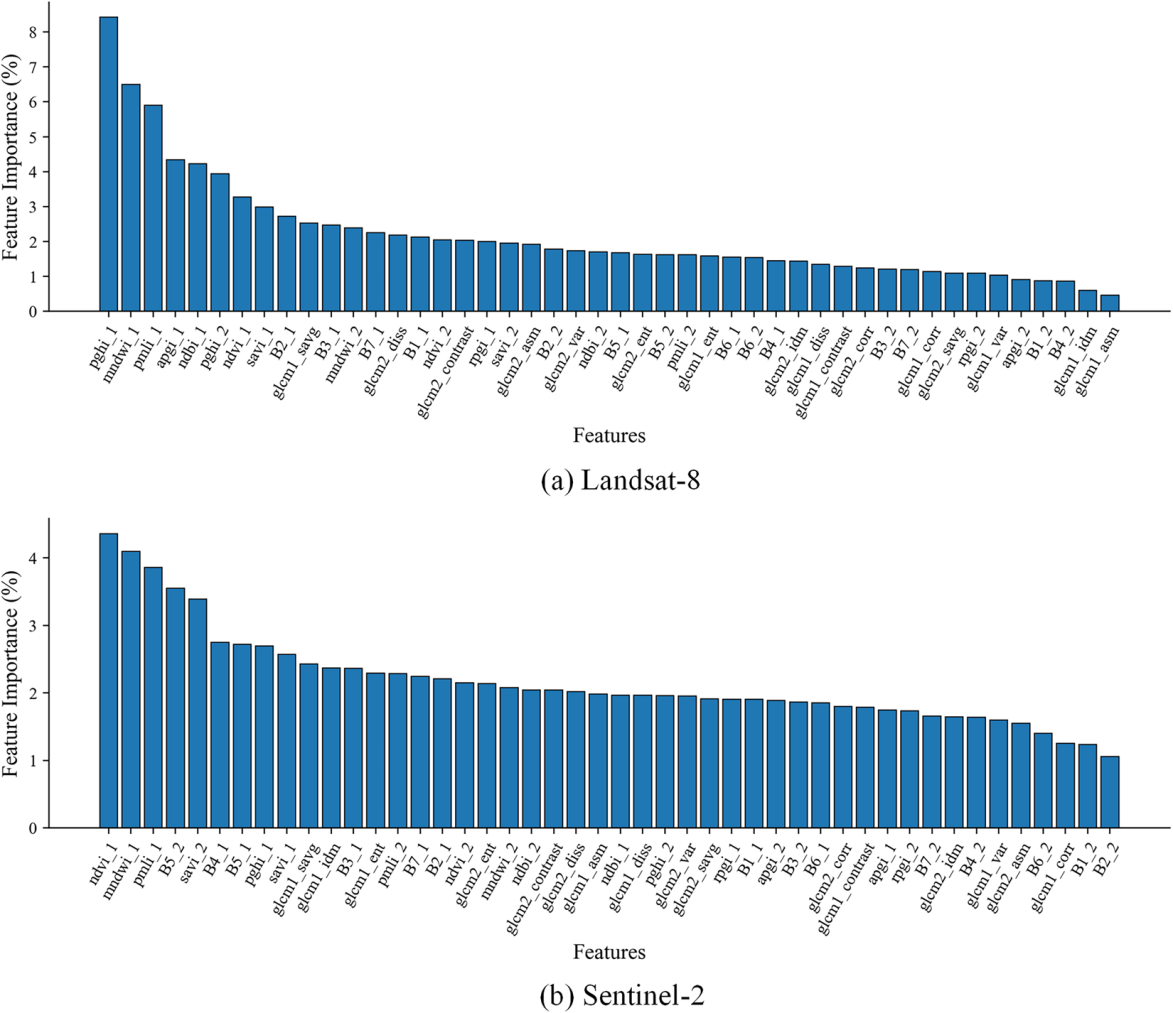
Feature importance derived from random forest. Note.* The suffix “1” denotes features from the first temporal phase, and “2” denotes features from the second temporal phase.

For feature construction, we used the GEE platform to obtain multi-source long-term imagery, from which spectral and texture features were extracted to build a multi-dimensional feature space (Table 1). The selection of spectral indices and texture features in this study was based on previous related studies and our preliminary experimental results^19,30,45,46^. To further improve the interpretability of feature selection, we also conducted a Random Forest feature importance analysis in a representative study area (Fig. 5). The results showed that, although the feature-importance rankings differed somewhat between Landsat-8 and Sentinel-2 owing to differences in spatial resolution, spectral configuration and acquisition timing, both spectral indices and texture features played important roles in PMFs identification. Among these features, vegetation indices such as NDVI^47^ and SAVI^48^ were mainly used to characterize crop growth status. NDBI^49^ was used to improve the separability between PMFs and building rooftops, whereas MNDWI^50^ was mainly used to reduce interference from water bodies. PMLI^13^ and plastic-cover-related indices, including APGI^51^, PGHI^52^, PGI and RPGI^53^, were used to enhance the identification performance of PMFs. Meanwhile, texture features further captured the spatial structural differences between PMFs and surrounding land-cover types. Meanwhile, for a better separation of PMFs to other land covers, these selected features were extracted from dual-temporal imagery (i.e., *film-on* and *film-off* stages).

**Table 1:** Feature dataset.

Category		Selected Features
Spectral bands	Landsat-5 & Landsat-7	*B1 (Blue), B2 (Green), B3 (Red), B4 (NIR), B5 (SWIR1), B7 (SWIR2)*,
	Landsat-8	*B2 (Blue), B3 (Green), B4 (Red), B5 (NIR), B6 (SWIR1), B7 (SWIR2)*,
	Sentinel-2	*B2 (Blue), B3 (Green), B4 (Red), B8 (NIR), B5 (RE1), B6 (RE2), B7 (RE3), B8A (RE4), B11 (SWIR1), B12 (SWIR2)*
Spectral index	*NDVI, NDBI, MNDWI, SAVI, APGI, PGHI, PGI, RPGI, PMLI*	
Texture index	*ASM, Contrast, Correlation, Variance, IDM, SAVG, Entropy, Dissimilarity*

### Random Forest algorithm

RF is an ensemble-based classification method that builds multiple decision trees using bootstrap sampling and integrates their outputs through majority voting or probability averaging. This ensemble strategy improves classification stability and reduces the risk of overfitting associated with individual trees. At each node split, RF selects a random subset of features, introducing randomness at both the sample and feature levels, thereby enhancing model diversity and generalization. Owing to its ability to handle high-dimensional features and its robustness to noise and outliers, RF has been widely applied in remote sensing image classification^12,30^. In this study, RF was employed as the core classifier to generate long-term PMFs maps of Northeast China from 1985 to 2025. Dual-temporal imageries (i.e., *film-on* and *film-off* stages) were used to derive spectral and textural features, and partitioned modeling was conducted within 0.5° × 0.5° grid cells. The RF classifier was configured with 150 decision trees and 4 candidate features per split, while all other parameters were set to be default values.

### Training and validation data

In this study, 1° × 1° grids were constructed across the study area, and the GLC_FCS30D dataset was used to filter and retain only those grids containing cropland (Fig. 1). PMFs mapping was then carried out within these grids using a partitioned modeling approach. This strategy improved classification efficiency through block-wise parallel processing and enabled independent image selection within each 0.5° × 0.5° grid. It also helped mitigate challenges such as the short *film-on* window of PMFs visibility and unusable imagery due to cloud, thereby ensuring continuity in the PMFs mapping results.

**Fig. 6:**
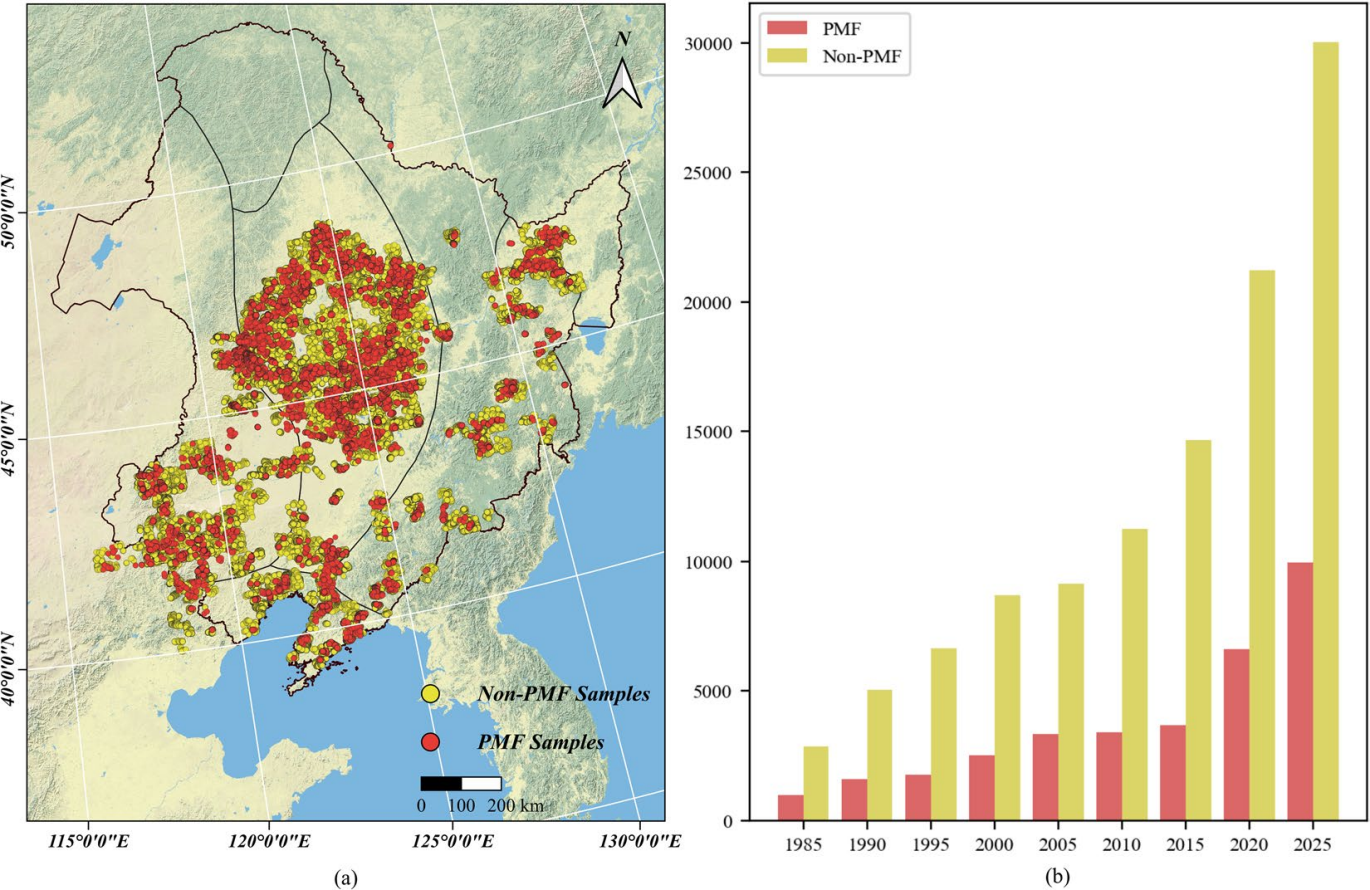
Spatial distribution of PMFs and non-PMFs training samples. Note.* The samples shown in (**a**) represent the aggregated sample set compiled from multiple years during the entire study period from 1985 to 2025. Projection: Albers Equal Area Conic projection.

Given the vast extent of Northeast China and the spatial heterogeneity of PMFs, sampling was performed by uniformly selecting samples within each grid cell. Two sample categories were defined, including PMFs and non-PMFs. Sample labeling was conducted through visual interpretation, supported by both Google Earth Pro, PlanetScope historical imagery, and field survey data. Since non-PMFs regions exhibit greater spectral heterogeneity and cover much larger areas than PMFs, the number of non-PMFs samples was set to be 2-3 times more than that of PMFs. In addition, samples were collected for nine representative years spanning the last four decades, yielding more than 100,000 samples in total (Fig. 6b). Multi-temporal images (i.e., *film-on* and *film-off*) were included to improve the reliability of visual interpretation for sample collection, resulting in a high-quality sample database. To ensure transparency and reproducibility, we also provide maps of the spatial distribution of training samples for both PMFs and non-PMFs classes (Fig. 6a).

### Post-processing

Since the initial PMFs classification results inevitably witnessed scattered isolated pixels and noise (i.e., salt-and-pepper effects), post-processing was applied to further improve PMFs mapping performance. Following previous studies^29,30^, we implemented the SieveFilter method on the GEE platform, which removes isolated pixels or patches smaller than a predefined threshold while preserving the distinct boundaries of PMFs. The selection of SieveFilter thresholds (3, 5, or 7 pixels) was adaptively determined based on the spatial resolution of the imagery to balance noise suppression and detail preservation. For 30-m Landsat data, a conservative threshold of 0 or 3 pixels was utilized to prevent the omission of genuine and fragmented PMFs regions. Conversely, for 10-m Sentinel-2 data, “salt-and-pepper” noise often consists of multi-pixel clusters. Therefore, a larger adaptive threshold range (3-7 pixels) was used. This resolution-dependent strategy could eliminate isolated artifacts while preserving the spatial morphological integrity of PMFs.

## Data Records

This proposed dataset, PMF-NEChina-40D, provides the first long-term maps of PMFs in Northeast China from 1985 to 2025. PMFs map files are organized by year (1985, 1990, 1995, 2000, 2005, 2010, 2015, 2020, 2025), while each yearly directory contains PMFs rasters named by a grid index, enabling grid-based retrieval using the accompanying grid-index Shapefile (*.shp). All PMFs maps are stored in GeoTIFF (*.tif) format at 30-m (Landsat series) and 10-m (Sentinel-2) spatial resolutions, with binary encoding (1 = PMFs, 0 = non-PMFs). The dataset is released under an open-access license and is publicly available on figshare^39^, allowing users to perform clipping, statistics, and re-analysis as needed.

## Technical Validation

This study evaluates the PMFs mapping performance using both qualitative and quantitative approaches. For the qualitative assessment, we adopted visual interpretation to examine spatial plausibility and visual consistency. For the quantitative assessment, we derived confusion metrics from randomly sampled validation points and computed a series of metrics including user’s accuracy (UA), producer’s accuracy (PA), F1 score, and overall accuracy (OA). Besides, comparisons with other datasets were not conducted because, to date, no other open-access PMFs products are available for Northeast China.

### PMFs mapping details

**Fig. 7:**
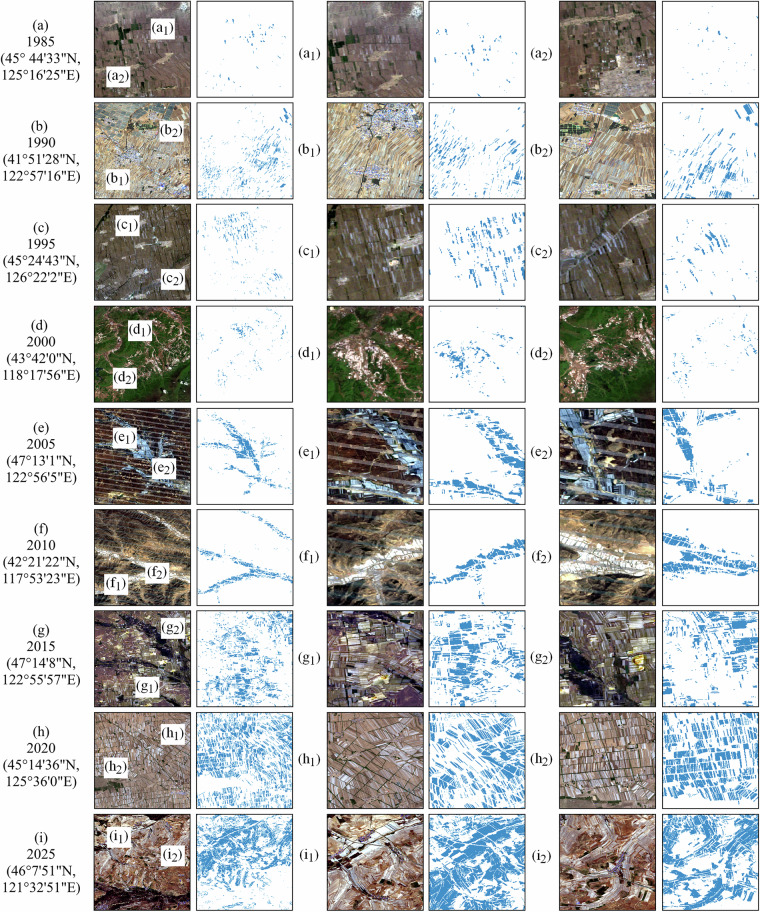
Classification details.

The details of PMFs mapping from different sensors are summarized as follows. (1) Landsat-5 TM (1985, 1990, 1995), shown in Fig. 7a–c. Despite the 30 m spatial resolution and the limitations of early-era data quality, PMFs distributions are still discernible at regional scale. (2) Landsat-7 ETM + (2000, 2005, 2010), shown in Fig. 7d–f. By using gap-filling with temporally adjacent imagery and post-processing, it could reduce the striping noise caused by the SLC-off failure, ensuring spatial continuity in the PMFs mapping results. (3) Landsat-8 OLI (2015), shown in Fig. 7g. Due to the higher signal-to-noise ratio and radiometric stability, it could improve the PMFs’ boundary delineation, resulting in more complete classification patches. (4) Sentinel-2 MSI (2020, 2025), shown in Fig. 7h,i. The 10-m spatial resolution allows for clear representation of PMFs boundaries, providing finer PMFs details in areas with small and fragmented parcels.

### Quantitative accuracy assessment

To further validate the reliability of the proposed PMFs dataset in Northeast China, we selected validation samples for each year using an equal allocation strategy, with the numbers of PMFs and non-PMFs samples kept consistent. In total, 15,000 PMFs and 15,000 non-PMFs validation samples were collected (Table 2). This balanced sampling design helps mitigate the influence of class imbalance on OA, making it more sensitive to PMFs classification errors. As shown in Table 2, the OA for all years exceeded 86%, with a maximum of 94.5%. The highest values of UA, PA, and F1 score reached 95.8%, 93.6%, and 94.7%, respectively. With improvements in satellite sensor and image quality, the accuracy metrics of PMFs classification show an upward trend. Using a sample-weighted approach, the average accuracies of the proposed dataset were as follows, UA = 93.40 ± 2.01%, PA = 90.89 ± 2.36%, F1 score = 92.11 ± 2.20%, and OA = 91.81 ± 2.29%. It demonstrates that the proposed dataset maintains high stability across different years and sensors, providing reliable data support for analyzing the spatio-temporal dynamics of PMFs over the past four decades in the Northeast China black soil region.

**Table 2:** PMFs mapping accuracy in Northeast China from 1985 to 2025.

Year	PMFs	Non-PMFs	Total	UA (%)	PA (%)	F1 score	OA (%)
*1985*	1,000	1,000	2,000	89.2	85.8	87.4	86.9
*1990*	1,000	1,000	2,000	90.4	87.1	88.7	88.3
*1995*	1,000	1,000	2,000	91.2	88.5	89.8	89.4
*2000*	2,000	2,000	4,000	92.0	89.3	90.6	90.2
*2005*	2,000	2,000	4,000	93.0	90.6	91.8	91.5
*2010*	2,000	2,000	4,000	94.0	91.8	92.9	92.6
*2015*	2,000	2,000	4,000	94.8	92.5	93.6	93.4
*2020*	2,000	2,000	4,000	95.5	93.2	94.3	94.1
*2025*	2,000	2,000	4,000	95.8	93.6	94.7	94.5
	*Weighted average*			*93.40* ± *2.01*	*90.89* ± *2.36%*	*92.11* ± *2.20%*	*91.81* ± *2.29*

### Comparison with official statistics and previous PMFs maps

**Fig. 8:**
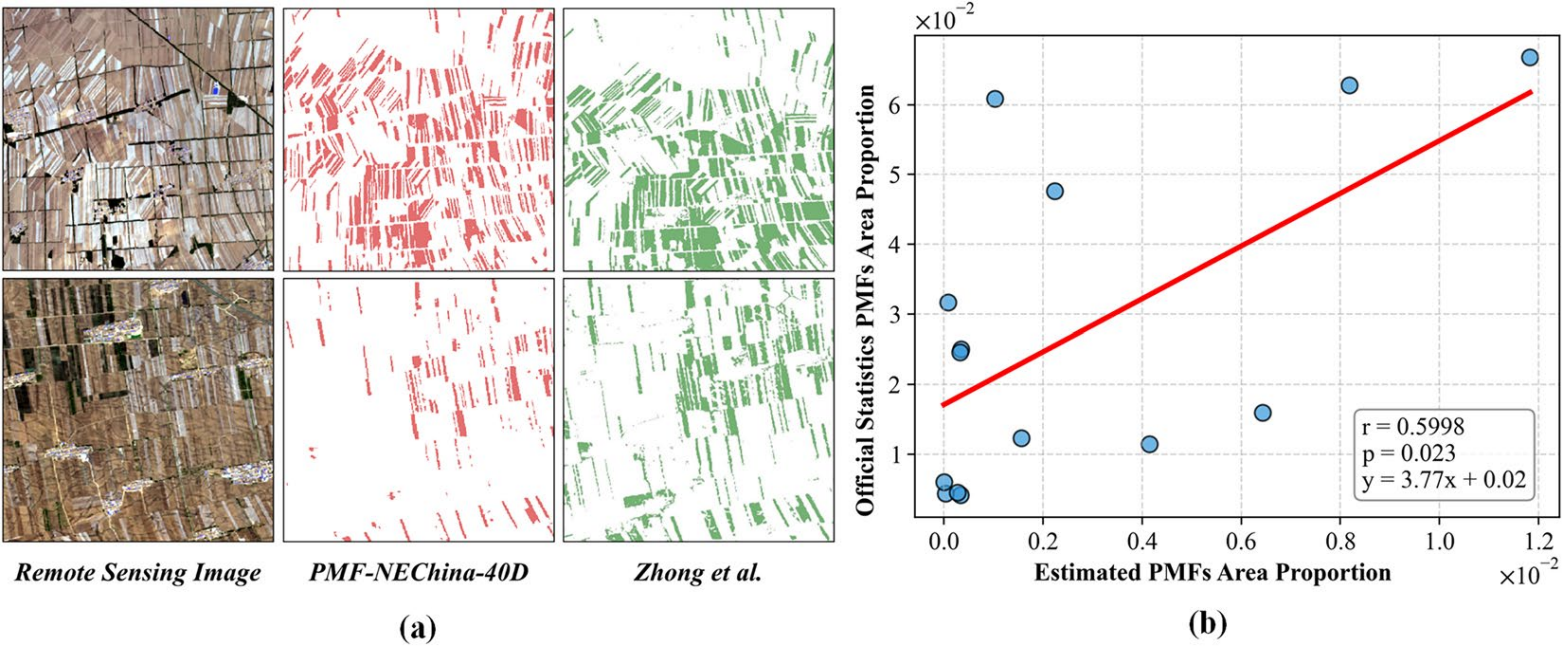
Indirect validation of PMF-NEChina-40D. (**a**) Visual comparison between PMF-NEChina-40D and the PMFs mapping results reported by Zhong *et al*. (2025); (**b**) Relationship between RS-derived PMFs proportion and official agricultural statistics at the municipal level.

Given the lack of publicly available long-term PMFs datasets for Northeast China for direct quantitative comparison, we conducted an indirect validation of PMF-NEChina-40D using municipal-level official agricultural statistics (https://www.stats.gov.cn/sj/). Considering data completeness and spatial consistency, 2020 was selected as the benchmark year because relatively more municipal-level statistics on PMFs were available for that year, covering a total of 14 prefecture-level cities. To reduce the influence of differences in administrative-unit size and improve data comparability, we normalized both the RS-derived and officially reported PMFs areas using cropland area from the authoritative Third National Land Survey (https://gtdc.mnr.gov.cn/), and then calculated and compared the PMF-to-cropland area ratios for each prefecture-level city. The results showed a clear positive correlation between the RS-derived and officially reported PMFs proportions (Fig. 8(b); r = 0.60). In addition, we referred to representative regional PMFs mapping results reported in previous studies^35^ and compared them with the PMF-NEChina-40D results in selected study areas. As shown in Fig. 8(a), both products captured the spatial distribution characteristics of PMFs in Sentinel-2 imagery. Comparatively, PMF-NEChina-40D places greater emphasis on precision in the mapping strategy to ensure a more reliable delineation of PMFs spatial distribution.

In addition, several measures were adopted in the PMFs mapping task to further ensure the accuracy and reliability of the proposed dataset.

#### Control of satellite image quality

It should be noted that PMFs mapping performance is highly dependent on image quality. On the GEE platform, we selected images with cloud cover less than 20%. Meanwhile, based on the phenological characteristics of PMFs, we focused on two key periods, including spring (*film-on*) and summer (*film-off*) to minimize confusion with greenhouses, bareland, clouds, and haze.

#### Quality control of training and validation samples

To improve sample quality, we resorted to both field survey and multi-source remote sensing data for sample data collection, including multi-temporal Landsat and Sentinel-2 data, very high resolution historical images from both Google Earth Pro and PlanetScope.

#### Partitioned modeling

Given the large extent, landscape heterogeneity of Northeast China, and computational constraints of GEE, we organized data using 1° × 1° grids and implemented classification at the 0.5° × 0.5° grid level. This partition strategy could increase parallel processing efficiency, while improving model generalization capability in heterogeneous areas.

#### Post-processing of PMFs mapping results

To further improve PMFs maps, we applied the SieveFilter method on the GEE platform to reduce salt-and-pepper noise in the initial PMFs maps without significantly altering the PMFs boundaries.

## Usage Notes

The proposed dataset, PMF-NEChina-40D, is provided in GeoTIFF format at 30 m (Landsat) and 10 m (Sentinel-2) resolutions for nine representative years from 1985 to 2025. Users can directly visualize and analyze the data using common GIS and remote sensing software such as QGIS or ArcGIS. Users may apply resampling or reprojection to align the dataset with other geospatial products such as cropland maps, land cover datasets, climate records, or soil property maps.

### PMFs density distribution

**Fig. 9:**
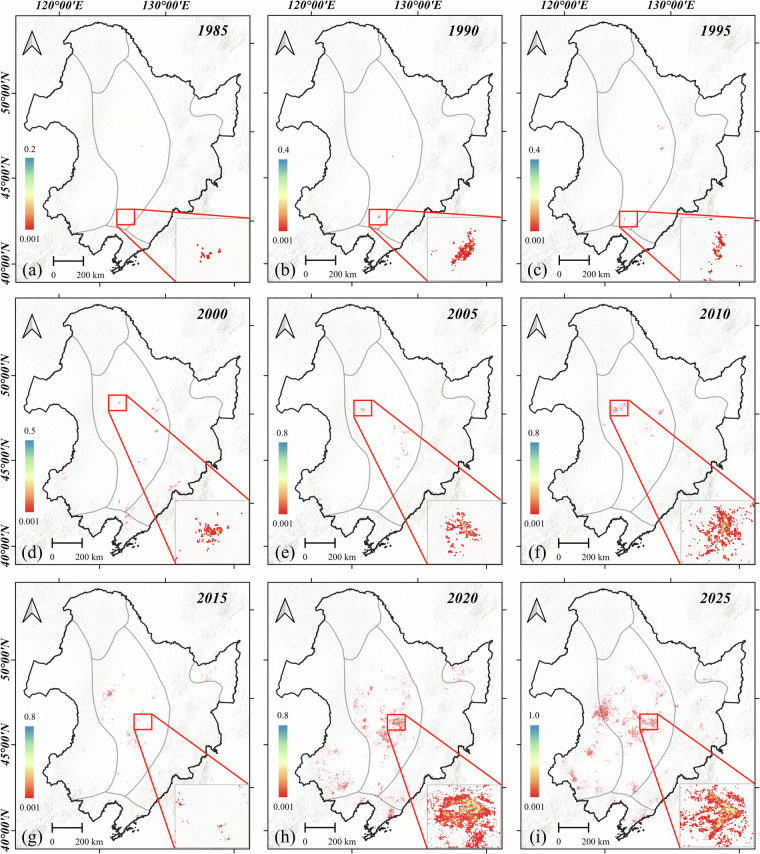
Spatio-temporal distribution of PMFs in Northeast China over the past four decades. (Projection: Albers Equal Area Conic projection).

**Fig. 10:**
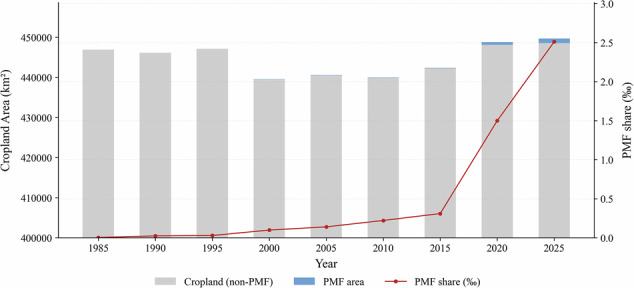
Cropland extent and PMFs share in Northeast China (1985–2025).

We generated PMFs density maps at 0.01° resolution to better capture their spatio-temporal heterogeneity and fine-scale variations (Fig. 9). Compared to raw pixel-wise classification, the density maps could clearly reveal inter-annual spatial patterns of PMFs. Figure 9 indicates that PMFs exhibit a pronounced increase over the past four decades in Northeast China. Specifically, from 1985 to 2010, PMFs’ distribution was sparse and patchy, whereas from 2015 through 2025, it shows multi-nodal expansion across the region. Accordingly, both PMFs mapped area and spatial footprint increased over time. We further quantified temporal changes (1985-2025) in cropland area and in the share of cropland occupied by PMFs across Northeast China. The results show that from 1985 to 2015 the PMFs share remained low (<0.3‰), followed by a marked increase while by 2025, PMFs occupied approximately 2.5‰ of the total cropland area (Fig. 10). All area estimates were computed using the Albers Equal Area Conic projection. By 2025, PMFs had expanded not only in total extent but also in spatial coverage, occurring across nearly all agro-climatic zones of Northeast China. These results demonstrate that the proposed dataset could support fine-scale spatial analyses and long-term trend assessments, providing a flexible benchmark dataset to investigate the spatio-temporal evolution of mulching agriculture in Northeast China black soil region.

### PMFs county-level distribution

**Fig. 11:**
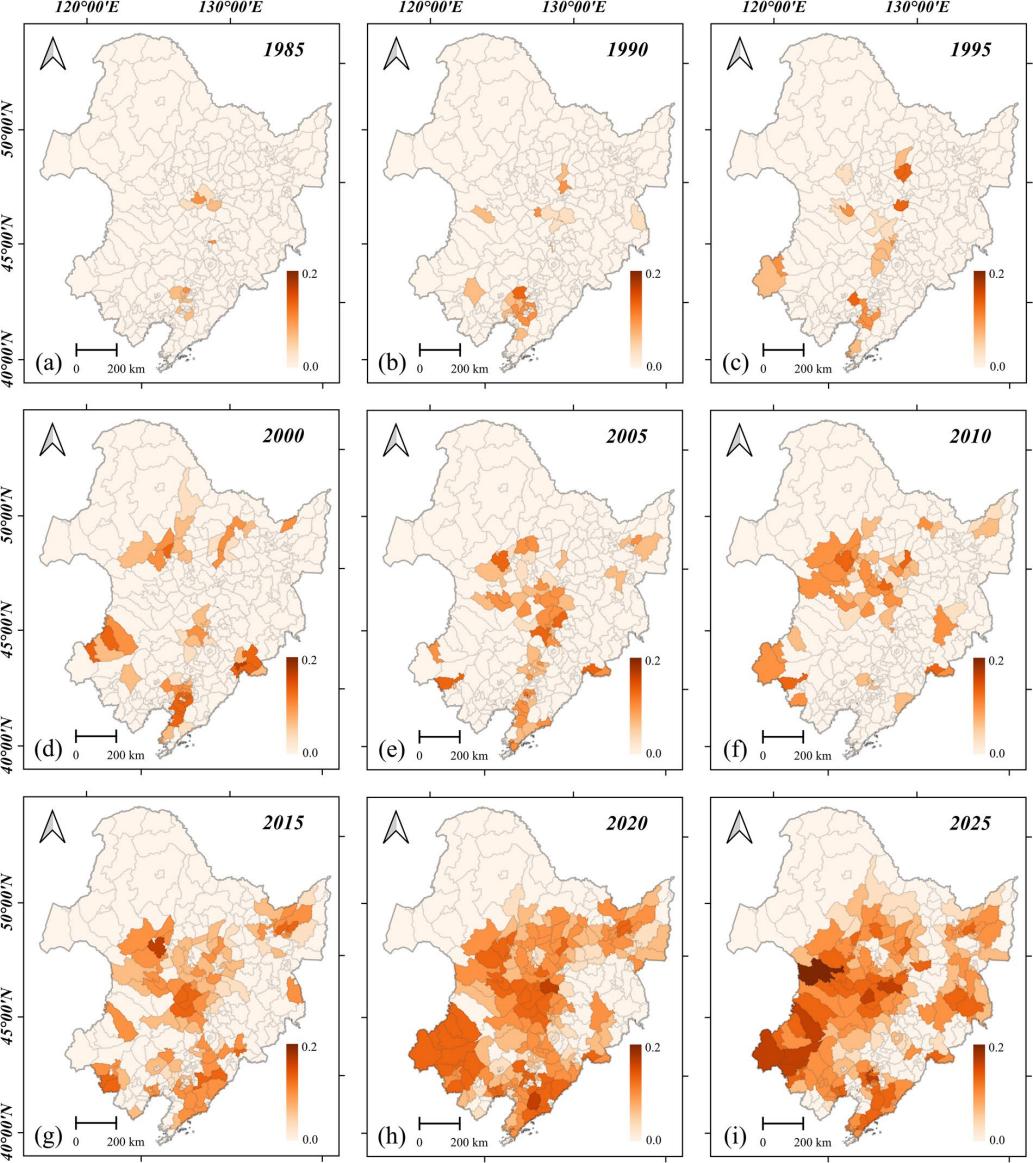
County-level distribution of PMFs in the past four decades. (Projection: Albers Equal Area Conic projection).

County-level statistics of PMFs area show that during 1985-1995, PMFs coverage was generally low, with more than 90% of counties below 0.01%. Only several counties in northern Liaoning, western Jilin, and southern Heilongjiang witnessed a high PMFs ratio of 0.01–0.1%. By 2000-2010, several counties exceeded 0.1% in PMFs ratio, while counties with more than 1% coverage of PMFs appeared. In recent years (2015–2025), high-coverage PMFs regions expanded quickly, with an increasing number of counties surpassing 1% by 2025. Figure 11 indicates that the proposed PMFs dataset can be flexibly aggregated to county level to reveal long-term spatio-temporal patterns and to support cross-regional comparisons.

Above all, the proposed dataset is suitable for diverse applications, including monitoring plastic pollution risks, evaluating ecological and environmental impacts, supporting agricultural policy-making such as black soil conservation strategies, and assessing agricultural non-point source pollution. All data are openly available and can be readily integrated with other environmental and agricultural datasets for reuse in interdisciplinary studies.

### Sources of uncertainty

It should be noted that the PMFs area estimated by the PMF-NEChina-40D dataset is systematically lower than official agricultural statistics. This discrepancy primarily originates from the following factors: First, our classification strategy prioritizes high user’s accuracy (precision), meaning that some highly fragmented or mixed PMF pixels may be omitted. Second, this study specifically targets white or transparent plastic mulch, whereas official statistics typically encompass all types, including black film. Due to a lack of historical ground-truth samples, dark-colored films are difficult to reliably identify in optical remote sensing imagery. Third, statistical records generally represent cumulative annual plastic film usage, whereas our remote sensing results capture the spatial distribution during a specific peak mulching period. Fourth, plastic films applied in orchards, forested areas, or inside agricultural facilities (e.g., greenhouses) are generally invisible to optical sensors. Furthermore, inherent limitations in the quality of early satellite imagery may introduce additional uncertainties into the classification results. Consequently, the proposed dataset is best suited for analyzing the spatial distribution patterns and relative temporal dynamics of PMFs.

## Data Availability

The dataset in this study, PMF-NEChnia-40D, is publicly available on figshare via 10.6084/m9.figshare.30636665.v1. Each raster provides a binary classification of plastic-mulched farmlands (PMF = 1; non-PMF = 0), with datasets from 1985–2015 produced at 30 m resolution and those for 2020 and 2025 at 10 m resolution. Data for each year are stored in separate folders named by their corresponding 1° grid ID (1degID), and the classification results within each folder are saved as 0.5° × 0.5° GeoTIFF tiles (05degID) following the naming format Year_pmf_1degID_05degID.tif. A vector file of the 1° grid system (NEChina_1_Degree) is provided for indexing, containing the ID attribute representing the 1° grid ID (1degID). More details are included in the Supplementary file.

## Code availability

The GEE code developed for PMFs classification is publicly available at https://github.com/MrSuperNiu/PMF-NEChina-40D-GEE. The code is implemented in JavaScript within the GEE environment and incorporates all key procedures described in this study, including feature extraction, Random Forest model training, classification, and accuracy evaluation. The workflow is fully reproducible, and users may adapt the scripts to extend the framework to other regions or temporal periods.
